# Bridging Neurobiological Insights and Clinical Biomarkers in Postpartum Depression: A Narrative Review

**DOI:** 10.3390/ijms25168835

**Published:** 2024-08-14

**Authors:** Keyi Zhang, Lingxuan He, Zhuoen Li, Ruxuan Ding, Xiaojiao Han, Bingqing Chen, Guoxin Cao, Jiang-Hong Ye, Tian Li, Rao Fu

**Affiliations:** 1 Department of Anatomy, School of Medicine, Shenzhen Campus of Sun Yat-sen University, Sun Yat-sen University, Shenzhen 518107, China; zhangky36@mail2.sysu.edu.cn (K.Z.); helx28@mail2.sysu.edu.cn (L.H.); lizhen26@mail2.sysu.edu.cn (Z.L.); dingrx3@mail2.sysu.edu.cn (R.D.); hanxj7@mail2.sysu.edu.cn (X.H.); chenbq29@mail2.sysu.edu.cn (B.C.); caogx3@mail2.sysu.edu.cn (G.C.); 2 Department of Anesthesiology, Pharmacology, Physiology & Neuroscience, Rutgers, The State University of New Jersey, New Jersey Medical School, Newark, NJ 07103, USA; ye@njms.rutgers.edu; 3 Department of Gynecology and Obstetrics, The Seventh Affiliated Hospital, Sun Yat-sen University, Shenzhen 518107, China

**Keywords:** postpartum depression, neuroimaging, animal models, clinical biomarkers, neurobiology, amygdala, hippocampus, striatum, the medial prefrontal cortex

## Abstract

Postpartum depression (PPD) affects 174 million women worldwide and is characterized by profound sadness, anxiety, irritability, and debilitating fatigue, which disrupt maternal caregiving and the mother–infant relationship. Limited pharmacological interventions are currently available. Our understanding of the neurobiological pathophysiology of PPD remains incomplete, potentially hindering the development of novel treatment strategies. Recent hypotheses suggest that PPD is driven by a complex interplay of hormonal changes, neurotransmitter imbalances, inflammation, genetic factors, psychosocial stressors, and hypothalamic–pituitary–adrenal (HPA) axis dysregulation. This narrative review examines recent clinical studies on PPD within the past 15 years, emphasizing advancements in neuroimaging findings and blood biomarker detection. Additionally, we summarize recent laboratory work using animal models to mimic PPD, focusing on hormone withdrawal, HPA axis dysfunction, and perinatal stress theories. We also revisit neurobiological results from several brain regions associated with negative emotions, such as the amygdala, prefrontal cortex, hippocampus, and striatum. These insights aim to improve our understanding of PPD’s neurobiological mechanisms, guiding future research for better early detection, prevention, and personalized treatment strategies for women affected by PPD and their families.

## 1. Insights into Postpartum Depression: Clinical Characteristics

Postpartum depression (PPD) is a prevalent and debilitating psychiatric condition often inadequately recognized and investigated due to its multifaceted etiology and suboptimal therapeutic interventions [1]. Clinically, PPD features disturbances in sleep patterns, anxiety, irritability, diminished concentration, a pervasive low mood, a sense of being overwhelmed, feelings of guilt, and even suicidal ideation in severe cases [2]. In addition, PPD presents distinct symptoms, including disinterest in the infant, absence of maternal bonding, feelings of being a bad mother, heightened anxiety in proximity to the baby, and fear of harming the baby or oneself [3].

The diagnostic criteria for PPD closely resemble those of major depression disorder (MDD) in the 5th edition of the Diagnostic and Statistical Manual of Mental Disorders (DSM-5). These criteria consist of symptoms such as depressed mood, feelings of worthlessness or excessive guilt, insomnia, diminished interest or pleasure in activities, changes in body weight, fatigue or loss of energy, psychomotor agitation or retardation, decreased ability to concentrate, and recurrent thoughts of death or suicidal ideation. Diagnosis requires the presence of five or more of these symptoms occurring during pregnancy or within four weeks after childbirth. Nevertheless, ongoing debate surrounds extending the diagnostic window to the first six months postpartum, given the heightened risk for PPD during this period [4].

The 10-item Edinburgh Postnatal Depression Scale (EPDS), endorsed by the American Academy of Pediatrics and the American College of Obstetricians and Gynecologists [5], is proven to be effective and has been widely used for PPD detection [6]. A two-question screening tool and the Patient Health Questionnaire 9 are also recommended for screening purposes [5,7]. These scales should be employed alongside sensitive inquiries and a comprehensive evaluation of various factors, including personal and family history, marital relationships, and level of social support. There are three degrees of postpartum mood disorders, namely the “baby blues”, PPD, and postpartum psychosis (PPP) [8]. Notably, PPD can be misdiagnosed as postpartum blues, which is considered a milder form of depression or adjustment disorder resulting from the stressors associated with childbirth [9]. However, postpartum blues tend to begin 1 to 3 days after parturition, last 2 weeks at least, and sometimes continue to develop into PPD [10]. PPP, characterized primarily by delusions, grandiosity, hallucinations, and confusion, should not be mistaken with PPD because PPP is rare and can progress rapidly, putting the lives of the mother and her baby at risk [5,11]. Therefore, an accurate diagnosis requires careful consideration of the clinical presentation and thorough assessments of the patient’s symptoms.

The spectrum of depressive manifestations associated with PPD varies from mild transient symptoms to more severe psychotic depressive disorders, as the DSM-4 describes. Utilizing EPDS scores, which are indicative of depression severity, PPD can be categorized into three distinct classes. With a mean EPDS score of 10.5, women in class 1 exhibit the mildest symptoms, followed by those in class 2 with a mean score of 14.8, and class 3 with a mean score of 20.1. Class 3 PPD is the most severe and substantially linked to many characteristics, such as suicidal thoughts, heightened anxiety, the development of symptoms during pregnancy, bad mood, and obstetric complications [12]. To treat PPD requires a combination of basic management, psychotherapy, and pharmacotherapy.

### 1.1. Basic Management and Psychotherapy

The practical treatment of PPD necessitates a multidisciplinary approach that prioritizes the safety and health of both the mother and infant. According to stepped care management, improving self-care and enhancing sleep protection and social support, as well as a moderate amount of exercise, lay the basis for treating all postpartum depressed women [5]. Moreover, psychotherapy is another essential approach. Cognitive behavioral therapy exerted significant positive effects on PPD women in both the short and long term [13]. Interpersonal psychotherapy is valid for improving depressive mood and demonstrates strong effectiveness in both individual and group treatment [14], but the optimal time for intervention should be between 4 and 12 weeks [15]. According to a meta-analysis, both therapy strategies show similar efficacy to each other when compared with other methods of psychological interventions, including client-centered counseling, support groups, and general practitioner management [16]. Repeated transcranial magnetic stimulation is also an efficacious, acceptable, and well-tolerated therapy [17].

### 1.2. Pharmacotherapy

When conventional interventions and psychotherapy fail to yield adequate responses, or in cases of severe PPD, medication should be considered. Regarding the use of antidepressants and anxiolytics, a tradeoff between their potential harm to breastfed infants and risks linked to patients’ abrupt discontinuation of necessary PPD treatments is indispensable. Though many selective serotonin reuptake inhibitors (SSRIs), serotonin-norepinephrine reuptake inhibitors (SNRIs), tricyclic antidepressive agents (TCAs), and benzodiazepines have access to breastmilk, infant plasma concentrations of these antidepressants are sometimes undetectable or very low, and adverse effects on the newborn, such as sedation, irritability, and diarrhea, are found only in a few cases [18,19,20], so the risk–benefit ratio could be in favor of breastfeeding depending on the situation. PPD guidelines in many countries agree on the compatibility of postpartum pharmacotherapy with breastfeeding, with a combination of clinical follow-ups and replacement with shorter elimination half-life and better-responded drugs [21]. 

Given the established relationship between sex hormones and brain function or affective disorders [22,23], interventions involving estradiol and progestin represent another potential approach for managing PPD. However, conflicting results regarding the efficacy of transdermal [24,25] and sublingual 17β-estradiol [26] therapy underscore the need for further randomized controlled trials with adequate participant numbers to establish their validity. Recent advancements in pharmacotherapy have introduced neuroactive steroids such as brexanolone [27] and zuranolone (SAGE-217) [28] as effective PPD treatments, which are approved by the U.S. Food and Drug Administration (FDA). These compounds act as positive allosteric modulators of GABA_A_ receptors, inhibiting inflammatory responses triggered by toll-like receptors 4 and 7 and the activity of enzymes within the kynurenine pathway [29,30]. Their approval represents a significant milestone in the development of neurosteroid replacement therapy for PPD.

In summary, it is significant for physicians to acknowledge the mentioned clinical characteristics, including symptoms, diagnosis, classification, and feasible treatments. However, given that PPD is a psychiatric disorder relating brain, an insight into the altered structure and chemicals via imaging tools and biomarkers is indispensable.

## 2. Multimodal Perspective of Neuroimaging Studies on PPD

Neuroimaging tools, such as functional magnetic resonance imaging (fMRI, including task- and resting-state fMRI), positron emission tomography (PET), and magnetic resonance spectroscopy (MRS), have already provided a wealth of potentially helpful information about MDD. These tools are also promising in PPD studies, demonstrating functional or structural alterations that could assist in diagnosis and prognosis (for review see [31,32]). Given the focus of many published studies on MDD, it is crucial to explore and compare neuroimaging findings in PPD.

### 2.1. fMRI Studies

#### 2.1.1. Task fMRI

Task fMRI is a powerful noninvasive tool that provides high-quality visualization of brain activity locations in response to sensory stimulation or cognitive functions [33]. Although fewer fMRI studies have focused on PPD compared with MDD, several findings highlight similarities and differences in neural activity between these types of depression (for review see [34]). For example, Laurent and Ablow observed reduced orbitofrontal cortex activity in depressed mothers in response to joyful infant faces, contrasting with increased activity in mothers with lower depressive symptoms [35]. This finding is consistent with the observation that people with MDD show less activity in the same brain area when exposed to sad-face stimuli [36]. 

The anterior cingulate cortex (ACC) helps process reward information and monitor outcomes [37]. A recent study found that brain activity patterns in the ACC are reduced in people with treatment-resistant MDD [38]. Similarly, a task fMRI study found less activity in this brain area in PPD patients when they were processing crying infants and upset facial expressions [39]. These abnormalities in the ACC might serve as potential imaging markers for depression.

However, the amygdala’s response in PPD is more complex. Amygdala activity increased in depressive mothers when viewing unfamiliar smiling infants, unlike in MDD and nondepressive mothers [40,41]. Conversely, another study found that unmedicated postpartum depressive symptoms correlated with decreased right amygdala activity in response to threat-related linguistic stimuli [42], differing from the heightened amygdala responsivity seen in unipolar depression patients [43]. 

#### 2.1.2. Resting-State fMRI

Unlike task fMRI, which measures brain activity during specific tasks, resting-state fMRI looks at brain networks when the person is at rest. These studies focus on evaluating the change in different brain regions’ connections (functional connectivity), the similarity of brain activity in nearby regions (regional homogeneity or ReHo), and changes in brain volume. It has been found that connectivity between the posterior cingulate cortex and the right amygdala is significantly disrupted in depressed mothers [44]. These brain regions are important parts of the default mode network, which is involved in memory, imagination, and other passive activities [45]. This network often shows different connectivity levels in MDD studies [46,47]. This disruption might help explain mental disorders in PPD patients and could serve as a potential imaging marker for both MDD and PPD. The study also documented decreased connectivity in the dorsomedial prefrontal cortex, dorsal ACC, and orbitofrontal cortex in PPD patients [48].

Besides changes in connectivity, patients with PPD show higher ReHo values in the right hippocampus and left precuneus, and lower ReHo values in the right insula and left dorsolateral prefrontal cortex [49]. ReHo measures how similar brain activity is in neighboring areas, with higher values indicating hyperactivity and lower values indicating hypoactivity in those regions [50,51]. 

Recent studies have also found increased gray matter volume in the left dorsolateral prefrontal cortex, orbitofrontal cortex, and right precentral gyrus in people with PPD. Researchers suggest this increase might be related to abnormal parenting behaviors, impaired executive function, and reduced stress tolerance [52]. Overall, these findings suggest that changes in activity and connectivity among the above in cortex structures are closely linked to PPD.

### 2.2. PET-CT

PET techniques are utilized to assess brain metabolism and detect alterations in protein expressions, such as receptor density, ligand transporters, and enzymes, under MDD (for review see [53]) and PPD conditions. PET is also used to evaluate drug occupancy, radiotracer competition, and the release of endogenous neurotransmitters. PET-CT studies suggest that critical molecules such as monoamine oxidase A (MAOA), dopamine receptors, and serotonin-1A receptors have diagnostic potential for PPD. Using 11C-harmine PET, independent studies have identified a significant increase (13–22%) in MAOA density in the ACC and prefrontal cortex (PFC) of PPD patients [54], and a 34% average increase in the ACC, PFC, hippocampus, thalamus, caudate, putamen, midbrain, and anterior temporal cortex of MDD patients [55]. Thus, MAOA, a key enzyme in monoamine metabolism involved in depression, might serve as a common imaging biomarker. 

Additionally, a 7–8% decrease in the availability of D2 and D3 dopamine receptors in the ventral striatum has been reported in postpartum women with unipolar depression, associated with both depression and postpartum status [56]. This finding aligns with previous research on the hypofunction of the dopamine system in MDD [57,58]. Abnormalities or dysregulation in the serotonergic system are crucial in forming MDD [59]. MDD individuals showed about a 26% decline in serotonin 1A receptor (5-HT1AR) in the mesiotemporal cortex [60], and significantly reduced postsynaptic binding of 5-HT1AR in the left lateral orbitofrontal cortex, medial temporal cortex, and subgenual ACC has also been observed in PPD [61], indicating another similarity that major depression shares with its subtype. Altogether, these findings suggest that disturbances in the homeostasis of monoamine neurotransmitter systems may be a key neurobiological alteration associated with the cortex of PPD patients.

### 2.3. MRS

MRS detects the radio frequency electromagnetic signals emitted by atomic nuclei within molecules, enabling the measurement of specific chemical concentrations in the brain and facilitating the kinetic analysis of dynamic neurochemical processes [62]. A study documented reduced metabolites in the left dorsolateral prefrontal cortex, specifically in the glutamate complex (Glx, comprising glutamate and glutamine) and N-acetyl aspartate (NAA) of PPD patients compared with healthy controls [63]. This finding is highly relevant to a recently published meta-analysis, which highlighted significant decreases in NAA concentrations within the frontal lobe in first-episode depression and chronic MDD patients [64]. Both findings illuminate NAA’s role as a potential biomarker for imaging diagnosis and treatment response, suggesting potential similarities between PPD and MDD, the latter of which is being extensively investigated.

Additionally, elevated glutamate levels were observed in the medial prefrontal cortex (mPFC) of unmedicated PPD women, with no changes in other metabolites like NAA and total creatine (t-Cr) [65]. However, a meta-analysis by Moriguchi found significantly lower Glx levels in the mPFC of medicated MDD patients compared with healthy controls, but not for glutamate or glutamine levels [66], while Merkl found decreased glutamate concentrations [67]. Given that postpartum hormone swings can lead to emotional disorders by regulating glutamate levels in the brain [68], this distinction between MDD and PPD might offer clues about their unique mechanisms or be due to different gender compositions in the studies. In the pregenual ACC and occipital cortices, there were no significant differences in gamma-aminobutyric acid to creatine ratios (GABA+/Cr) between PPD patients and controls [69]. Overall, these results suggest that mPFC hyperactivity may be linked to PPD, but further research is needed. 

Regarding neuroradiological examinations, the scientific community is working on optimizing contrast media to address potential risks for patients and their babies during the critical postpartum stage. Innovative biodegradable nanoshells, such as those using hyaluronic acid as a carrier, are being developed to enhance the stability and tolerance of contrast agents for MRI, PET, and other applications. This could make imaging safer, more tolerable, and potentially more accurate for postpartum patients. However, further clinical trials are needed to advance these developments from research to practical use [70].

## 3. Serum and Plasma Biomarkers

In PPD research, various biomarkers have been identified as potential indicators, though their reproducibility across studies is limited, possibly due to the diverse clinical manifestations in PPD patients [71]. These biomarkers fall into three main categories: hormones (both reproductive and stress-related), inflammatory markers (indicating immune system involvement), and biochemical markers (providing a molecular footprint of the disorder). 

### 3.1. Hormones

#### 3.1.1. Reproductive Hormones

The association of reproductive hormone swings during pregnancy, childbirth, and postpartum events with neurophysiological changes is well documented [72]. Research indicates that individuals predisposed to PPD have a heightened sensitivity to the mood effects of gonadal steroids, suggesting different hormonal responsiveness [73]. However, the literature varies on the role of sex hormones in PPD pathophysiology, likely due to small sample sizes in some studies, emphasizing the need for larger-scale research [74]. Lower concentrations of allopregnanolone, a neurosteroid metabolite of progesterone, have been linked to PPD, suggesting a neurobiological basis [75]. Additionally, decreased plasma oxytocin levels are associated with a higher risk of PPD during both gestational and postpartum phases [76]. This evidence highlights the complexity of hormonal influences and the need for further comprehensive research to fully understand these relationships.

#### 3.1.2. Stress Hormones

Stress hormones indicate hypothalamic–pituitary–adrenal (HPA) axis responses, with corticotropin-releasing hormone (CRH) frequently associated with PPD in clinical studies. Quantitative analyses have established a substantial correlation between EPDS scores and serum CRH concentrations [77]. Emerging research suggests that the prognostic efficacy of CRH may be time-dependent. One study showed that depressive symptoms at three months postpartum correlated significantly with increased CRH levels during mid-gestation, but this did not extend to symptoms at six months postpartum, indicating a temporal restriction in CRH’s predictive value for PPD [78]. Besides serum CRH, placental CRH, a vital coordinator of parturition, lactation, and maternal behaviors [79], has also been recognized as a potential biomarker for PPD prognosis and detection [80]. Overall, these findings suggest CRH is a promising biomarker for PPD, but further research is required to delineate its diagnostic value.

### 3.2. Inflammatory Cytokines

The etiological link between neuroinflammation and PPD has been reinforced through extensive research. Elevated concentrations of interleukin (IL)-1 have been observed during the early postpartum stage, specifically on the 14th and 28th days, in PPD patients compared with those in euthymic states, indicating a link between depressive symptoms and higher IL-1 levels during the first month after delivery [81]. Another study focusing on postpartum African American populations revealed a significant positive correlation between serum IL-6 and IL-1β concentrations and the severity of depression [82]. Further refined analysis using adjusted Least Absolute Shrinkage and Selection Operator (LASSO) logistic regression pinpointed an upsurge in specific inflammatory mediators, including IL-18, fibroblast growth factor 23 (FGF-23), tumor necrosis factor ligand superfamily member (TRANCE), hepatocyte growth factor (HGF), and C-X-C motif chemokine 1 (CXCL1) [83]. Collectively, these advancements pave the way for inflammatory cytokines to become early predictors of PPD.

### 3.3. Biochemical Markers 

Besides the two main categories mentioned above, some other chemicals were proposed to have diagnostic potential. For instance, a diminished serum zinc concentration (but not magnesium) might contribute to an increased risk of developing PPD [84]. Insufficient vitamin D intake or lowered serum levels of 25-hydroxylated vitamin D have also been reported in PPD pathology [85]. Studies also elucidated an enhanced density of MAOA [54], augmented homocysteine concentrations [86], and low docosahexaenoic acid status after pregnancy [87], thereby suggesting their potential roles in the predisposition to PPD. Moreover, alterations in neurotransmitter and neuropeptide levels, including decreased plasma serotonin and neuropeptide Y levels, along with increases in norepinephrine and substance P levels [88], as well as elevated β-endorphin concentrations [89], have further been associated with PPD. 

This accumulating body of evidence demonstrates the complexity of PPD etiology. To comprehensively assess these biochemical parameters as potential biomarkers or therapeutic targets, an understanding of their biological mechanisms is essential.

## 4. Biological Mechanisms Involved in the PPD

### 4.1. A Unified Model of PPD

**Figure 1:**
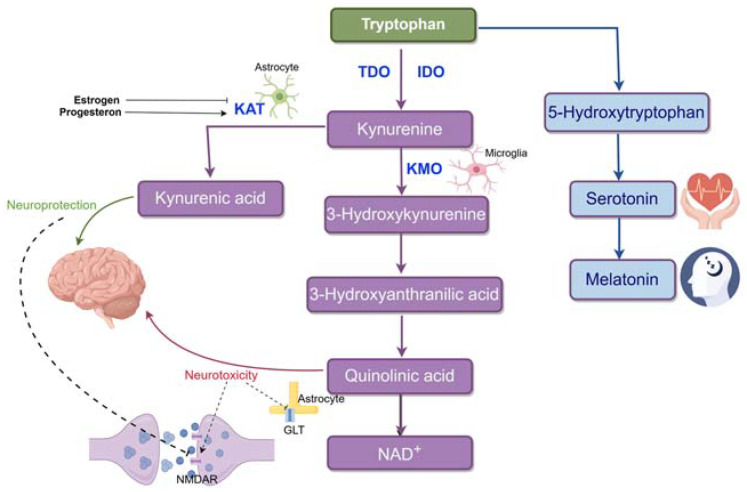
Tryptophan metabolism in the brain. Tryptophan is mainly metabolized through the kynurenine pathway (95%) and less so through the serotonin pathway (<5%). In the kynurenine pathway, tryptophan is converted to kynurenine by IDO (in the brain) or TDO (in other tissues). This pathway splits into two branches: the KMO branch, where KMO (primarily in microglia) produces 3HK, 3HAA, QUIN, and NAD+, and the KAT branch, where KAT (mainly in astrocytes) converts kynurenine to KYNA. KYNA is neuroprotective, while QUIN is neurotoxic, with distinct effects on brain function.

Understanding PPD requires knowledge of the serotonin and kynurenine metabolic pathways, both involving tryptophan metabolism. Indoleamine 2,3-dioxygenase (IDO) and tryptophan dioxygenase (TDO) enzymes mediate tryptophan conversion into kynurenines, with IDO primarily active in the brain and immune cells, and TDO in other tissues. Imbalances in the equilibrium between the serotonin and kynurenine pathways can significantly impact various medical conditions, including depressive disorders. Serotonin’s multifaceted role in mental pain regulation and weight maintenance, as well as being a precursor for melatonin, underscores its relevance in this context [90]. A biased conversion of tryptophan to kynurenine over serotonin may increase susceptibility to PPD. Pathophysiological disruptions to this equilibrium will be detailed in subsequent sections. Please refer to Figure 1 for a visual depiction of these pathways.

The kynurenine pathway has two branches. In one branch, kynurenine is metabolized into 3-hydroxykynurenine (3HK) by kynurenine-3-monooxygenase (KMO), yielding 3-hydroxyanthranilic acid (3HAA), quinolinic acid (QUIN), and ultimately, nicotinamide adenine dinucleotide (NAD+). QUIN exhibits neurotoxic effects by activating the NMDA receptor and stimulating presynaptic glutamate release [91]. It also impedes glutamate uptake into astrocytes by blocking glutamate transporters. Within this branch, 3-HK acts as a neurotoxic agent, promoting neurotoxicity through neuroinflammation and lipid peroxidation-associated autoimmune responses [92]. In the other branch, kynurenine is converted into kynurenic acid (KYNA) by kynurenine aminotransferase (KAT). KYNA is generally recognized as neuroprotective, competitively inhibiting ionotropic glutamate receptors at high concentrations and reducing activity at the glycine co-agonist site of the N-methyl-D-aspartate receptor (NMDAR). The divergent impacts of these metabolic branches arise partly from their distinct glutamatergic activity at the NMDAR [93]. 

As an essential neurotransmitter, serotonin is produced in several tissues, including brain serotoninergic neurons [94]. The brain’s serotonin concentration is partially influenced by the plasma tryptophan levels since tryptophan enters the brain through neutral amino acid transporters. A range of 5-HT receptor subtypes are activated when serotonin, which is produced from tryptophan by the enzyme tryptophan hydroxylase, is released into the synaptic cleft after being stored presynaptically. The serotonin transporter facilitates the reuptake of serotonin, which is either recycled for further release or degraded by monoamine oxidase and eliminated in urine.

Genetic research further supports the hypothesis that the tryptophan-serotonin metabolism pathway plays a part in PPD. There is a strong correlation between the long allele carriers of the serotonin transporter, the 5-HTTLPR gene, and PPD. The 5-HTT gene polymorphism primarily consists of two types of gene length polymorphism: 5-HTTLPR polymorphism (classified as L or S allele according to the presence of 44 base pairs in the promoter region) and STin2VNTR polymorphism (determined by the number of tandem repeats in the second intron). The effects of the 5-HTT gene and 5-HTTLPR gene are usually discussed with environmental factors. Studies conducted by Divya Mehta demonstrated that polymorphisms in 5-HTT serve as a predictor only when pregnant women have undergone adverse life events, which is to say the S allele amplifies the effect of negative life experiences on depression symptoms, particularly in the latter stages of the postpartum period [95]. There is also a study finding that, though there is no significant association between 5-HTTLPR polymorphism and PPD, the seasonal differences in childbirth and 5-HTTLPR polymorphism have a joint influence on the severity of PPD [96]. 

### 4.2. Endocrine Dynamics of PPD

During pregnancy, human bodies often experience dramatic changes in hormone levels, especially reproductive hormones, which are thought to be associated with the onset and progression of mental disorders. Since a variety of hormonal alterations can have an impact on the pathways involved in tryptophan metabolism, we can also discuss endocrine levels and their related genes as influential factors of the unified model which centers on tryptophan metabolism. It’s important to note that there are always interrelationships between reproductive hormones and that reproductive hormones impact HPA axis functions and are simultaneously impacted by the HPA axis.

#### 4.2.1. Estrogen 

Estrogen levels increase dramatically before delivery, reaching 1000 times their baseline values, then drop quickly after delivery with substantial individual differences. Animal models have demonstrated that withdrawal of estradiol induces depression-like behaviors and that estradiol treatment can reverse that kind of behavior in ovariectomized mice [97]. Cytokines have chronic effects on the brain and lead to cytokine-induced depression. Studies have found that estrogen is linked to inflammatory changes, positively correlated with IL-6, and negatively correlated with kynurenine [98]. By reducing the expression of KynA and its neuroprotective effects, estrogen, and its derivatives may interfere with the KP balance, thereby intensifying the expression of the QUIN branches and their neurotoxic effects [3]. It is worth mentioning that estradiol disulfate is a much more potent inhibitor of KAT enzymes than estrone sulfate, estradiol, and estradiol 3-sulfate.

#### 4.2.2. Progesterone 

Progesterone levels, like estrogen, rise steadily during pregnancy and drop below the baseline after delivery. Nevertheless, to the contrary, progesterone acts as an activator of KynA and an anti-inflammatory agent, negatively correlated with the inflammatory factor IL-1β and several kynurenine pathway metabolites, including nicotinamide and quinolinic acid [98]. Progesterone also interacts with the immune system, regulating T-cells, which play a crucial role in the termination of the inflammatory response [99].

#### 4.2.3. Oxytocin 

Oxytocin (OXT) is well known for its regulating function for emotion, stress, and mother–baby relationships, as well as its involvement in delivery and lactation. Studies have shown a negative correlation between OXT during pregnancy and a positive screen score of 10 or higher on the EPDS, suggesting a higher risk for the development of PPD. This implies that mothers with lower plasma OXT concentrations during pregnancy are more susceptible to depressive symptoms [76]. The secretion of OXT is promoted by behaviors like breastfeeding, touching, and partner interactions. The absence of these behaviors may weaken the protective effect of OXT. As a protective factor, OXT reduces inflammation and the activity of the HPA stress axis, which are two activators of KP. OXT reduces the HPA stress axis mainly through the GABA signaling pathway, primarily via an increase in GABA_A_ receptors. KP also influences the secretion of OXT since NAD+ is a precursor of cADPR. Activation of CD38 by various types of receptors triggers the formation of cADPR [100]. In chronic inflammation, there may be an imbalance of CD38-cADPR, leading to a decline of OXT at the expense of a more robust immune response.

OXT and OXTR genes are important regulators of nursing behavior in mothers, which has a connection with the depression-like behaviors of the postpartum period. An SNP in the OXTR gene and methylation state was investigated to be associated with PPD [101]. In a study, polymorphisms in OXT rs2740210 interacted with early-life adversity to predict variation in breastfeeding duration since variants in OXT rs2740210 moderated the effects of early adversity on depression. Mothers who experienced early adversity displayed increased depression and reduced breastfeeding only if they carried the CC genotype of OXT rs2740210 but not if they possessed the AA/AC genotypes [102]. Intriguingly, PPD triggered by early life adversity and OXT polymorphisms may result in a shorter breastfeeding duration of the mother to the offspring, which may make the daughter more susceptible to PPD, forming a vicious circle of the heredity of PPD in the family [103]. On top of that, the interaction of quality of early care and OXT gene polymorphism on maternal mood at 6 months postpartum has also been demonstrated [104].

Studies revealed that epigenetic mechanisms, particularly in the context of DNA methylation, involve the alteration of the gene associated with PPD. Key research points to the OXTR gene, indicating that the methylation levels of this gene are associated with serum estradiol and the allopregnanolone-to-progesterone ratio, suggesting an intricate relationship with estrogen’s role in modulating the oxytocin system [105]. It has been established that estrogen enhances OXTR transcription in the brain and uterus, where poor maternal experiences might lead to increased methylation at estrogen response elements, disrupting the interaction of the estrogen receptor complex. A notable study found that women with PPD had increased OXTR DNA methylation among those with the rs53576 GG genotype [101]. In contrast, the connections between the 5-HTT gene’s epigenetic mechanisms and PPD are less understood, with one study (Devlin et al., 2010) showing associations with Edinburgh Postnatal Depression Scale scores and SLC6A4 promoter methylation during pregnancy [106].

#### 4.2.4. Stress Hormones

It has been widely acknowledged that stress and adverse life events can break the balance of the HPA axis and alter its function. In clinical diagnosis, in addition to interviews and self-rating questionnaires, the HPA axis hormones are often considered to provide a profile of the endocrine system. Many studies have dived into the three main hormones of the HPA axis (CRH, ACTH, and cortisol), which act as a biological timer of pregnancy [107]. The dramatic rises of CRH, ACTH, and cortisol levels are led by the additional CRH secreted by the syncytial cells in the human placenta from the 7th week of pregnancy. CRH is typically only released from the hypothalamus into portal circulation, but it is also released from the placenta during gestation. During pregnancy, the positive feedback loop of cortisol to placental CRH and the negative feedback loop of cortisol to CRH generated by the hypothalamus jointly influence the levels of stress hormones. PPD is linked to the desensitization of HPA axis hormones during the restoration of these hormones, which occurs roughly 12 weeks after delivery. Nondepressed women lose the cortisol response to an acute stressor, suggesting that pregnancy buffers the physiological responses to stress and that high levels of CRH may impair the HPA response to psychosocial stress [108]. During the postpartum period, the HPA axis is exposed to extreme perturbations in the form of the acute withdrawal of pCRH due to the delivery of the placenta. There is a reduction in suppression in response to dexamethasone administration for at least five weeks following delivery, suggesting that women exhibit a delayed ACTH response to an external CRH challenge during the first postpartum months [109].

In addition to the physiological changes in stress hormones due to pregnancy, the unique social stressors experienced during pregnancy like unstable partner relationships, concerns about the offspring’s development, and anxiety over body changes can also affect the levels of stress hormones. Among these, the most prevalent and inescapable one is sleep deprivation. Regarding maternal sleep needs, nursing infants demand continuous care that often interrupts the mother’s sleep cycle, challenging her energy conservation. The disruption of sleep homeostasis is usually followed by an increase in hypothalamic–pituitary–adrenal (HPA) axis activity, leading to a surge in circulating stress hormones. Animal studies indicate maternal adaptations, such as instances where mother rats can simultaneously nurse and sleep [110], a tactic not immediately translatable to humans, who often experience impaired sleep and relationship issues with their infants when affected by PPD. Clinical evidence shows considerable intersections between disturbed sleep and PPD [111], suggesting further exploration into sociocultural influences and differences in stress thresholds between humans and animals might illuminate divergent responses to PPD-related sleep deprivation [112].

The pathogenesis of diseases is a complex and multilevel process of scientific research. The theoretical hypothesis forms the basic framework of the cause and development path of the disease. However, to put these theories into practice and verify their effectiveness, the translational tool of animal models is needed.

## 5. Laboratory Animal Models of PPD

**Figure 2:**
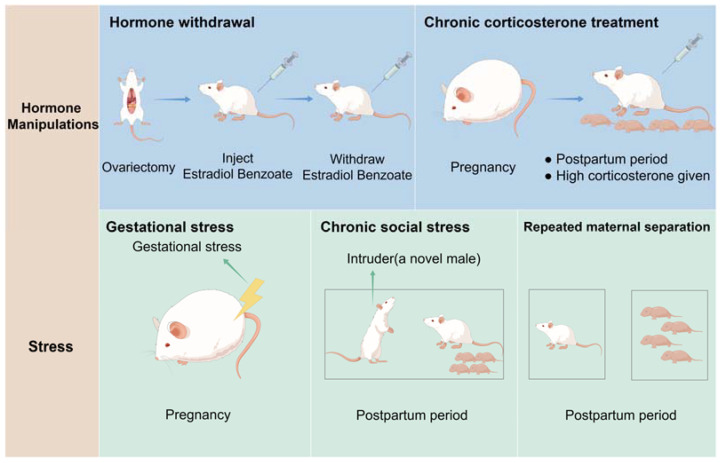
Laboratory Animal models of PPD: (1) Hormone withdrawal models. Ovariectomized female rats were injected with a low dose of estradiol benzoate and a high dose of progesterone dissolved in sesame oil daily for 16 consecutive days. From day 17 to 23, the dose of estradiol was increased to mimic the levels observed in pregnancy. From day 24 to 27, which is considered the postpartum period, mice only received vehicle (sesame oil) daily. (2) Chronic corticosterone treatment model. Dams were injected with high CORT during the postpartum period (postpartum day 2–24). (3) Gestational stress model. Depression is induced by exposing pregnant female mice to gestational stress from days 10 to 20 during pregnancy. (4) The chronic social stress model. A novel male was placed in their home cage for 1 h each day from days 2 to 16 of lactation, resulting in depressive behaviors. (5) The repeated maternal separation model. Depression is induced by separating the mothers from their pups for periods that last 3–6 h daily during the first 1–3 weeks postpartum.

Animal models, particularly rodents, are invaluable for studying the etiology, pathophysiology, and treatment options for PPD due to their physiological similarity to humans and the ease of experimental manipulation. Mir et al. categorize rodent PPD models into two main groups: hormone manipulation and stress exposure, which reflect pregnancy-related changes or simulate known PPD risk factors, respectively [113]. Particularly, the hormone manipulation models include the Hormone Withdrawal Model and the Chronic Corticosterone Treatment Model, which simulate depressive-like states through hormonal changes. On the other side, the stress exposure models encompass the Gestational Stress Model, which induces chronic stress during pregnancy, the Chronic Social Stress Model (CSSM), which uses daily social defeat to mimic social conflict, and the Repeated Maternal Separation Model, which isolates mothers from their offspring to study stress effects on maternal behavior (see Figure 2). The following list details these rodent models as used in recent PPD-related studies.

### 5.1. Hormone Withdrawal Model

Estrogens (estradiol, estriol, and estrone) and progesterone increase significantly during pregnancy, decline rapidly after childbirth, and return to prepregnancy levels by the fifth postpartum day [114]. These rapid hormonal changes may be linked to PPD [23]. Therefore, studies of depressive-like behavior in rodents during the postpartum period often use the hormone withdrawal regimen as a model. Galea developed the hormone-simulated pregnancy (HSP) model in rats. In this model, ovariectomized female rats receive a large dose (4 mg) of progesterone dissolved in 0.1 mL sesame oil daily for 16 days, along with a modest dose (2.5 μg) of estradiol benzoate (EB). From day 17 to day 23, the estradiol dose is increased to 50 μg to mimic pregnancy levels. During the postpartum period (days 24–27), the pregnant group receives only a vehicle (0.1 mL sesame oil) daily. Depressive-like behaviors are then assessed using the forced swim test after three days of hormone withdrawal. Findings indicate that female rats undergoing estradiol withdrawal exhibit more depressive-like behaviors, including reduced struggling and increased immobility [115].

It should be noted that this model has its limitations. For instance, oxytocin and prolactin do not increase as they would during the postpartum period, and some maternal behaviors, such as pup grooming and nursing, cannot be evaluated using this model [113]. Additionally, because the females are ovariectomized, the model cannot directly assess the impact of parturition and its effects on the offspring [116]. Despite these limitations, the hormone withdrawal model successfully reproduces some phenotypes observed in PPD. Therefore, it remains a well-established and valid approach for researching novel pharmacological treatments.

### 5.2. Chronic Corticosterone Treatment Model

Perinatal dysregulation of the HPA axis is a significant contributing factor to PPD [109]. Building on this, Brummelte et al. developed a model that replicates the hypercortisolism seen in major depression [117]. Their study involved administering high doses of corticosterone (CORT) at 40 mg/kg from postpartum days 2 to 24, resulting in decreased body weight and reduced maternal care, enhancing depressive-like behavior similar to that in human mothers with depression. A notable advantage of this model is its ability to minimize variability associated with different stressors, providing greater control over hormone levels than other physical or social stress models [113]. This controlled environment enhances the reproducibility and reliability of experimental outcomes, facilitating a more precise investigation into the mechanisms underlying PPD and its associated hormonal dysregulation.

### 5.3. Gestational Stress Model

This model aims to induce chronic stress during pregnancy to investigate the potential development of PPD. In one model devised by J.W. Smith, randomly selected dams underwent daily 1 h sessions of restraint stress by placing them in plexiglass restrainers from days 10 to 20 of pregnancy. The findings revealed that dams subjected to gestational stress exhibited depression-like behaviors and provided less frequent and intense maternal care [118]. Additionally, Zoubovsky et al. recently introduced a model of “variable psychosocial stress” during gestation in mice. This protocol involved various stressors, including foreign object exposure, rat scent exposure, a 30-degree cage tilt, removal of bedding, exercise on a shaker, and regular bedding changes (each lasting 2 h, performed twice daily with a minimum 2 h break in between) during the day, as well as overnight cage mate alteration, damp bedding, and continuous light exposure from day 6.5 to day 16.5 of pregnancy. The study observed depressive-like, anhedonic, and anxiogenic-like phenotypes during the initial postpartum period [119]. These studies indicate that long-term exposure to social psychological stress is a major contributor to PPD, whether during pregnancy or the postpartum period. Moreover, these animal models provide valuable tools for investigating emotional changes associated with gestation and lactation.

### 5.4. Chronic Social Stress Model (CSSM)

It posits that various factors such as sociodemographic variables, psychosocial stress related to pregnancy, depression, risky health behaviors, prepregnancy medical and psychiatric conditions, pregnancy-related ailments, and birth results may contribute to PPD development. Nephew and Bridges (2011) introduced this model to encapsulate aspects of PPD etiology. In contrast to the gestational stress model, the chronic social stress model employs a chronic social defeat paradigm wherein a novel male is brought into the home cage of the CSS group daily for one hour during lactation (days 2–16) [120]. This model has been shown to diminish maternal consideration and increase maternal aggression toward the male invader, suggesting its utility in simulating depression-like behaviors in animals induced by social conflict. Repeated Maternal Separation Model: This model is based on the premise that maternal separation from offspring induces significant stress for the dam, impacting maternal behavior and emotional responses. Typically, maternal separation entails isolating mothers from their offspring for a duration of 3 to 6 h daily within the first 1 to 3 weeks after giving birth. Boccia et al. introduced maternal separation as a model of postpartum depression, demonstrating expanded fixed status in the Forced Swim Test (FST) and reduced maternal behavior after recurrent separations from the mother [121]. Unlike models employing physical or pharmacological stressors, maternal separation utilizes a mental and social stress regimen, mirroring the etiopathogenesis of PPD in humans. This approach closely mirrors the pathogenesis of the disease in humans [122]. The animal models mentioned above are illustrated in Figure 2.

On this basis, the study of animal models not only provides an intuitive understanding of the pathophysiological process of the disease but also allows the in-depth exploration of the mechanism of brain regions. Through detailed anatomical, neuroimaging, and behavioral analysis, researchers have been able to locate the role of specific brain regions in diseases at the macro and micro levels, which undoubtedly enriches our understanding of the pathogenesis of diseases and provides a key basis for further clinical treatment strategies. 

## 6. Neuronal Mechanisms of PPD 

Several brain regions and complicated circuits are involved in the mechanisms of PPD, among which the PFC, amygdala, hippocampus, and striatum are widely studied. 

### 6.1. PFC 

PFC is linked to cognitive and emotion-regulation deficits resulting from chronic stress [123]. An increasing number of research associates PPD pathogenesis with PFC alterations, including changes in neurotransmitters, organelles, receptors, and inflammation. Erin et al. chose the validated and translationally relevant chronic mild unpredictable stress (CMUS) Wistar rat model and observed decreased mitochondrial function in the PFC using high-resolution respirometry, highlighting a potential role for mitochondrial function in postpartum health [124]. The medial PFC (mPFC)’s role in regulating rats’ postpartum maternal behaviors is further underlined (for review see [125]). In addition, the activation of 5-HT_2A_ receptors in mPFC by selective agonists disrupted maternal behaviors in a dose-dependent manner, thereby emphasizing the importance of these receptors in the normal expression of maternal behaviors [126]. Lastly, Benedetta et al. discovered that gestational stress in Sprague-Dawley rats led to postpartum depressive-like behaviors, accompanied by reduced density and altered morphology of dendritic spines in mPFC [127]. It illustrated how chronic stress induces PPD by impairing structural plasticity in mPFC.

### 6.2. Amygdala

The amygdala is a critical brain region that governs decision-making processes, autonomic functions, endocrine, and instinctual behaviors in response to the changing environment [128]. 

Neuronal plasticity includes structural changes such as dendritic spine density and functional alteration. It has become the focus of many studies concerning the amygdala. In Wistar rats, a postpartum drop in neuronal spine density within the amygdala was noted, indicating potential causes of emotional instability [129]. Using ovariectomized mice, Yang et al. established that postpartum estrogen reduction disrupts GABA_A_ inhibition in the basolateral amygdala due to a downregulation of estrogen receptor GPR30, thus impairing long-term depression induction and leading to anxiety-like behaviors [130]. This conclusion linked synaptic plasticity in PPD with perinatal fluctuation of ovarian hormones. Pantelis et al. found the behavioral impacts of acute allopregnanolone infusion into the brain were also restricted in the basolateral amygdala, in which PV interneurons uniquely expressed GABA_A_R δ subunit-containing receptors. It clarified the amygdala’s role in a novel molecular and cellular mechanism that mediates the well-established anti-PPD effects of neurosteroids [131]. Furthermore, the 18 kDa translocator protein (TSPO), a mitochondrial cholesterol transporter, is involved in PPD [132]. Li et al. showed the TSPO ligand ZBD-2 can alleviate depressive behaviors in ovarian steroid withdrawal mouse model by reverting specific glutamic receptor, GABA receptor, and 5-HT receptor expression and TSPO concentration in the basolateral amygdala back to the baseline [133]. It highlighted the important role of neurosteroid synthesis and suggested that its deficits in the amygdala may play a vital part in the pathology of PPD.

### 6.3. Hippocampus

The brain regions responsible for maternal behavior experience both structural and functional alterations throughout pregnancy and the postpartum period [134,135,136]. Gray matter volume is reduced during pregnancy in the hippocampus, which remains for at least two years after childbirth in first-time mothers contrasted with preconception [137]. The neurobiology of depression has been linked to it [138], and it is partially reversed in later postpartum [137,139,140]. However, what should be noted is that lower hippocampal gray matter volume was relevant to more positive maternal caregiving [141]. Hoekzema’s study concludes that a notable decrease in GM volume from prepregnancy to postpartum brain scans correlated with increased maternal attachment, self-reported ratings, and enhanced neural responses to infant faces [137].

Neuroplasticity in the hippocampus of rodents is diminished both during pregnancy and in the postpartum period [142,143]. The generation of new cells is a key component of hippocampal plasticity, presenting a potential avenue through which depression may impact plasticity within the hippocampus. In a study, depression-like rats established from the hormone withdrawal model had significantly lower levels of cell proliferation in the hippocampus [144]. Brummelte and Galea’s study has similar results. Dams treated with a high dose of CORT during the postpartum period showed more depressive-like behavior in the forced swim test and a reduction in hippocampal cell proliferation [117].

Regarding the plasticity of synapses and dendritic spines, the atrophy of apical dendrites of pyramidal neurons in the CA3 region of the hippocampus was significantly higher in pregnant rats with repeated constraint stress than in unstressed female rats or unmated female rats [145]. Besides, mice exposed to chronic restraint stress exhibited signs of depressive-like behavior, with an upregulation of NR1 expression and a downregulation of Akt/mTOR/GluR1 signaling. GluR1, a postsynaptic protein, is a subunit of AMPARs (ionotropic glutamate receptors) that are critical for the expression of plasticity and are responsible for rapid antidepressant effects [146].

Alterations in hippocampal plasticity may result from the change in the levels of several substances in the brain. As a critical molecule influencing the hippocampus’s maintenance and remodeling, reduced levels of brain-derived neurotrophic factor (BDNF) in the hippocampus are related to depression [147]. Tim Vanmierlo’s study showed that maternal stress during pregnancy reduced BDNF and p11 protein levels, but elevated p75NTR levels in the hippocampus of mouse mothers. BDNF receptor p75NTR induces apoptosis, and p11 regulated by BDNF may play a role in antidepressant action [148]. In addition, the separation of mother and child for 3 h/d during a puerperal period can reduce the level of nitric oxide and the activities of Na+ and K+ -ATPase in rat hippocampus [149]. Reduced levels of nitric oxide (NO) lead to decreased neural activity and synaptic plasticity in the hippocampus [150], and the reduced activity of Na+, K+ -ATPase has been identified as an important characteristic of depression in certain studies [151,152].

Additionally, compared with pregnant rats, postpartum rats with depressive-like behavior had lower hippocampal 3a and 5aTHP levels. Rats undergoing gestational stress had exhibited reduced levels of 3a,5a-THP in the hippocampus and a slight elevation in swimming duration These results suggest that 3a and 5a-THP in the hippocampus may be involved in the regulation of depressive behavior in female rats. It can be seen that BALB/c mice with depressive behavior had lower levels of 5-HT, neurotransmitters, and dopamine (during PREG) in the hippocampus [153].

### 6.4. The Striatum and Its Implications in PPD

Depression manifests primarily through low mood and a pervasive loss of interest and pleasure in life’s activities, known as anhedonia. The ventral striatum (VS) is pivotal in hedonic processing and reward reinforcement. Its function involves the regulation of dopamine and serotonin release, as well as the integration of limbic structures with prefrontal cortices [154].

It has been observed that mothers experiencing PPD show decreased striatum activation when exposed to positive stimuli, such as positive words [155] and faces [156], compared with nondepressed counterparts. Additionally, attenuated striatal reactions have been noted in PPD mothers when exposed to their infant’s cry [39]. Together, they emphasize the significance of the VS in upholding positive emotions and reward processing during the postpartum period for maternal–infant well-being.

The striatum plays a crucial role in the mesolimbic system, as it receives projections from dopaminergic neurons that originate in the ventral tegmental area (VTA) and project to the nucleus accumbens (NAc) [157]. Dopamine is crucial in neurodevelopment, including neuronal proliferation, migration, dendritic and axonal growth, synaptogenesis, and spinogenesis [158].

Studies on depressive behavior in animal models, such as BALB/c mice during pregnancy and the postpartum period, have revealed correlations between such behavior and decreased levels of 5-HT, dopamine, and norepinephrine in the striatum, particularly concerning caregiving behaviors [153]. Further research utilizing high-fat diet models of PPD has indicated diminished dopamine signaling in the NAc of postpartum rats [159], accompanied by reductions in dendritic length, branching, and spine density on medium spiny neurons in the NAc shell subjected to maternal stress [54].

Moreover, PET studies have demonstrated elevated levels of MAOA in the striatum of depressed postpartum women [54]. Given MAOA’s role in monoamine catabolism, increased levels of this enzyme could contribute to dopamine deficiency, thereby disrupting reward processing mechanisms in PPD patients. Overall, diminished striatum activity and impaired dopamine function appear to be associated with disrupted reward processing in PPD. These findings underscore the significance of comprehending the neurobiological foundations of PPD to develop targeted interventions for affected individuals.

## 7. Limitations

The keywords related to postpartum depression (PPD) were widely searched across multiple databases, including Web of Science, Google Scholar, and PubMed. This narrative review focuses on studies from the last 15 years, acknowledging its inherent limitations. Despite encompassing literature across epidemiology, brain imaging, biomarkers, therapeutics, animal models, and theoretical hypotheses, the lack of a systematic literature search may have omitted some crucial studies. However, the review offers valuable insights into PPD, despite its constraints. The field faces challenges in replicating findings from major depressive disorder (MDD) studies due to the complex nature of the nervous system and patient diversity.

## 8. Conclusions

**Figure 3:**
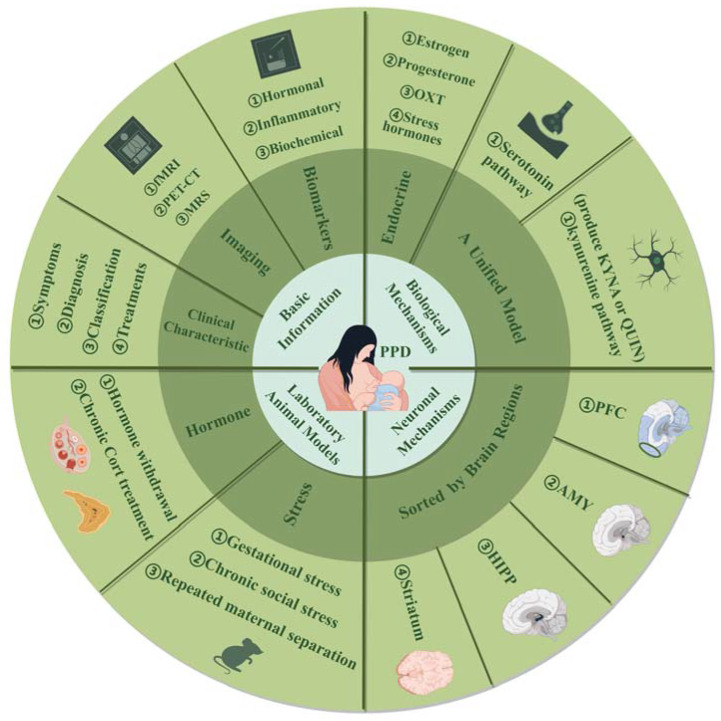
This review provides a comprehensive examination of postpartum depression (PPD) across four aspects: (1) Basic information, including clinical characteristics, brain imaging, and biomarkers, to offer an initial understanding. (2) Biological mechanisms, detailing PPD pathology with an emphasis on serotonin and kynurenine pathways, where kynurenine branches into neuroprotective KYNA and neurotoxic QUIN. (3) Laboratory animal models focused on hormone manipulation and stress, with model variations. (4) Neuronal mechanisms, involving key brain regions such as the PFC, AMY, HIPP, and striatum. Abbreviations: fMRI: functional magnetic resonance imaging; PET-CT: positron emission tomography-computed tomography; MRS: magnetic resonance spectroscopy; OXT: oxytocin; KYNA: kynurenic acid; QUIN: quinolinic acid; Cort: corticosterone; PFC: prefrontal cortex; AMY: amygdala; HIPP: hippocampus.

In summary, extensive research underscores the complex biological mechanisms underlying postpartum depression (PPD), involving reproductive and stress hormones such as estrogen, progesterone, oxytocin, and HPA-axis hormones, as well as tryptophan metabolism. Key brain structures—hippocampus, amygdala, striatum, and mPFC—show alterations in neuronal plasticity, reflecting intricate network changes. While animal models provide valuable insights into PPD, their generalizability is limited, and there is often a disconnect between preclinical findings and clinical application. To advance the field, future studies should focus on developing more diverse and comprehensive animal models that better represent the multifaceted nature of PPD. Integrating factors like stress, social dynamics, and genetic variability can enhance model relevance. Additionally, fostering closer collaboration between preclinical and clinical researchers will help bridge the gap between experimental results and real-world treatments. Adopting an integrated approach that encompasses neurobiological, psychological, and social dimensions will likely lead to more effective interventions and a deeper understanding of PPD (see graphic abstract in Figure 3).
